# Obtaining of Ni/NiO nanopowder from aqua solutions of Ni(CH_3_COO)_2_ ammonia complexes

**DOI:** 10.1186/s11671-015-0841-3

**Published:** 2015-03-29

**Authors:** Iryna Dulina, Tetyana Lobunets, Leonid Klochkov, Andrey Ragulya

**Affiliations:** Frantsevich Institute for Problems of Materials Science of National Academy of Science of Ukraine, Kiev, Ukraine

**Keywords:** Nickel ammine complexes, Nanopowders, Slit pore structure, Free carbon content, Monomodal particle size distribution

## Abstract

Ni/NiO nanopowders have been prepared by using thermal decomposition of aqua solutions of nickel acetate ammine complexes in air at the annealing temperature range of 300°C to 500°C, time of decomposition from 30 to 180 min, and ammonia content in initial complex 3.6 to 9.55 mol/mol Ni^2+^. Chemical composition of obtained powders has been characterized by chemical and thermal analysis. Phase analysis and particle size of powders have been investigated by X-ray diffraction method, transmission electron microscopy (TEM), and scanning electron microscopy (SEM). The powders’ pore structure has been determinated by low-temperature nitrogen adsorption method.

Products of decomposition were represented as agglomerates of nanoparticles of Ni, NiO, and hydroxy-containing precursors. Mean agglomerate size depended on ammonia content in initial complex, annealing temperature, and duration and has grown from 30 to 40 to 400 to 520 nm. Mean nanoparticle size of hydroxy-containing precursors was invariable with ammonia concentration in initial complex, annealing temperature, and duration and has grown 5 nm. Mean nanoparticle size of Ni depended on annealing temperature and has grown from 40 to 60 to 40 to 70 nm at temperatures 400°С and 500°С, respectively. Mean nanoparticle size of NiO increased with temperature rising from 5 nm at 350°С to 20 to 25 nm at 500°С.

## Background

Nickel powders are widely used as electrode materials in multilayered ceramic capacitors. The tendency of ceramic and electrode layers thinning to 100 to 200 nm that is used for increasing of capacitor dielectric capacity leads to the necessity of the powder size decreasing to 10 to 20 nm. At the same time, these powders used as electrode materials should fulfill the requirements for morphology, particle size distribution, ability to disperse in organic solvents, and impurity composition. Every one of these characteristics effects properties of conductive paste for screen printing, electrode layers obtained from it, and multilayered ceramic capacitor as a whole.

Size of solid particles, particle size distribution, and powder ability to disperse in organic solvents determinate viscosity, rheological properties [1,2], and sedimentation stability of the paste in the first place. In the second place, they effect continuity [3,4] and roughness [2] of the electrode. Particularly, in paper [2] it was determinated that paste which contains powders with monomodal particle size distribution has the minimal viscosity and permits to obtain electrode layers with minimal roughness. At the same time using of powders with dendrite structure [3] or high pores content in particle bulk [4] for paste preparing impairs evenness of electrode surface and leads to breaking of electrode surface after sintering.

Impurity composition effects paste viscosity and electrical properties of a multilayered ceramic capacitor. For example, Na and K presence decreases powder electroconductivity and the breakdown voltage of the capacitor [5,6]. Sulfur concentration in powder over 200 ppm leads to sizeable viscosity rising of electrode pastes for screen printing that complicates the process of capacitor manufacturing [7,8]. Carbon impairs powder conductivity and leads to increasing of the Schottky barrier because of eutectic melting of electrode layers under sintering temperature as a result of Ba/Ti/Ni alloy formation [9].

In usual, existing methods of powder synthesis do not take into account all or part of the demands on impurity composition, morphology, and particle size distribution of nickel powders which are used for production of multilayered ceramic capacitors.

Gas-phase methods such as thermal pyrolysis of nickel salt melts [10-15] and spray [16] and ultrasound [17-19] pyrolysis of aqueous solution of nickel salts in nitrogen or hydrogen-nitrogen atmosphere do not allow the obtaining of powder particles less than 160 to 190 nm because of the use of high temperatures of synthesis from 400°C to 1,400°C. Moravec et al. [20] obtained Ni/NiO nanopowder with particle size 10 to 50 nm by vapor deposition of nickel acetylacetonate in nitrogen and hydrogen-nitrogen atmosphere at the temperatures range 400°C to 500°C. But it can be noted that their own account of using of high temperatures of synthesis can lead to aggregate and dendrite formation during cooling of products that is inadmissible for electrode materials.

Liquid-phase methods are inadequate for obtaining of electrode materials because of the high content of impurities in powders [5,6]. Since the usual synthesis process comes from the formation of nickel hydroxide suspension [5,6,14,15,21-24], obtained powders contain a high concentration of sodium and potassium impurities. The use of tetra methyl ammonium and tetra ethyl ammonium instead of NaOH allowed the authors of papers [5,6] to decrease sodium content in nickel powder from 95 to 8.7 to 9.5 ppm but simultaneously led to an increase of carbon content. In addition, the decrease of the particle size of powders in liquid-phase methods can be achieved by the addition of dispersants - polar organic compounds [3,4,14,15,21,23,25-27] or ammonia or sodium salts of organic acids [22,26-28]; these lead to a rise of carbon in powder content from 1.5 to 4 wt. %. Another source of undesirable impurities for this group of synthesis methods is the use of reductive agents - polyethylene glycol [5,6,25], sodium formate [29,30], titanium (III) chloride [3,4], and NaBH_4_ [26,27].

Besides, complete or partial exchanging of Ni powder in the green electrode layer on NiO can result in electrode thinning during annealing in reductive atmosphere due to the powder volume decreasing [31]. In addition, this exchange can decrease BaTiO_3_ concentration in electrode which is added with the aim of balancing the sintering speeds of the electrode and dielectric layers [32,33] in the one hand and reduction of the dielectric capacity of the multilayered ceramic capacitor in the other hand. Thus, development of technology of Ni/NiO nanopowders obtaining a particle size of 20 nm and less and minimal impurity content has a great importance.

The use of unstable nickel compounds is a sufficiently promising method for synthesis of nanopowders of electrode materials with minimal impurity content. In this paper, a method of thermal decomposition of nickel acetate ammines in air was proposed. Ammines of nickel acetate are unstable compounds and decompose at relatively low temperatures. This fact permits the inhibition of particle size grown in the one hand. In the other hand, these compounds consist of ammonia which can reduce nickel ions to metal nickel at higher temperatures.

Thus, the paper is aimed at the investigation of the effect of annealing temperature and duration and ammonia content in initial complex on the powder composition and particle size.

## Methods

Ni/NiO nanopowders have been prepared by using thermal decomposition of aqua solution of nickel acetate ammine complexes in air. Complexes have been obtained by adding of nickel(II) acetate tetrahydrate in ammonia aqua solution. Ammonia content in initial complex was 3.6 to 9.55 mol/mol Ni^2+^. Obtained complexes in a porcelain crucible have been put in a muffle furnace heated to 300°C to 500°C and annealed in air atmosphere for 30 to 180 min.

In accordance with results of IR spectroscopy and thermal analysis (thermogravimetric (TG), derivative thermogravimetric (DTG), differential thermal analysis (DTA)) which have been presented in our previous papers [34-39], the full complex decomposition to Ni, NiO, and free carbon was not observed at experimental conditions. And the powders contained some amount of precursors that had complex composition and can decompose with the forming of metal and oxide at further heating to 350°C to 400°C. Because of this fact, element powder composition such as Ni, O, C, H, and N content do not permit estimating the completeness of complex decomposition and Ni and NiO yield at different synthesis conditions. In addition, precursor presence resulted in failure of direct analysis of NiO content by reduction powder in hydrogen atmosphere.

But combination of obtained results of IR spectroscopy, TG, DTG, DTA, and X-ray diffraction [34-38] allowed making the following conclusions:

1. Precursors can be divided as hydroxy-, carbonate-, and acetate-containing.
2. Full precursor decomposition is observed at temperatures higher than 120°C and less than 400°C.
3. In accordance with the results of IR-spectroscopy, residuals of organic compounds are too close to acetate ions. In addition, carbonate and acetate ions do not bond with Ni and decompose in the last stages.
4. Hydroxy-containing precursors can decompose with the forming of Ni and NiO. Acetate- and carbonate-containing precursors decompose with the forming of NiO mainly.

These conclusions permit the calculation of powders’ chemical composition by a combination of chemical and thermal analysis with the use of the following system of equations:

$$ \left\{\begin{array}{c}\hfill {m}_0={m}_{{\mathrm{H}}_2\mathrm{O}}+{m}_{{\mathrm{C}}_0}+{m}_{\mathrm{Ni}}+{m}_{\mathrm{Ni}\mathrm{O}}+{m_{\mathrm{Ni}{\left(\mathrm{O}\mathrm{H}\right)}_2}}_{.}+{m_{\mathrm{Ni}\mathrm{C}{\mathrm{O}}_3}}_{.}+{m}_{\mathrm{Ni}{\left(\mathrm{C}{\mathrm{H}}_3\mathrm{C}\mathrm{O}\mathrm{O}\right)}_2}.;\hfill \\ {}\hfill {m}_{120}={m}_{{\mathrm{C}}_0}+{m}_{\mathrm{Ni}}+{m}_{\mathrm{Ni}\mathrm{O}}+{m}_{\mathrm{Ni}{\left(\mathrm{O}\mathrm{H}\right)}_2-pr.}+{m}_{\mathrm{Ni}\mathrm{C}{\mathrm{O}}_3-pr.}+{m_{\mathrm{Ni}{\left(\mathrm{C}{\mathrm{H}}_3\mathrm{C}\mathrm{O}\mathrm{O}\right)}_2}}_{;}\hfill \\ {}\hfill {C}_{120}=\frac{m_{\mathrm{Ni}}+\frac{M_{\mathrm{Ni}}}{M_{\mathrm{Ni}\mathrm{O}}}\cdot {m}_{\mathrm{Ni}\mathrm{O}}+\frac{M_{\mathrm{Ni}}}{M_{\mathrm{Ni}{\left(\mathrm{O}\mathrm{H}\right)}_2}}\cdot m\mathrm{N}\mathrm{i}{\left(\mathrm{O}\mathrm{H}\right)}_2-pr+\frac{M_{\mathrm{Ni}}}{M_{\mathrm{Ni}\mathrm{C}{\mathrm{O}}_3.}}\cdot {m}_{\mathrm{Ni}\mathrm{C}{\mathrm{O}}_3}+\frac{M_{\mathrm{Ni}}}{M_{\mathrm{Ni}{\left(\mathrm{C}{\mathrm{H}}_3\mathrm{C}\mathrm{O}\mathrm{O}\right)}_2}}\cdot {m}_{\mathrm{Ni}{\left(\mathrm{C}{\mathrm{H}}_3\mathrm{C}\mathrm{O}\mathrm{O}\right)}_2}}{m_{120}};\hfill \\ {}\hfill {m}_{400}={m}_{\mathrm{Ni}}+{m}_{\mathrm{Ni}\mathrm{O}}+\frac{M_{\mathrm{Ni}}}{M_{\mathrm{Ni}{\left(\mathrm{O}\mathrm{H}\right)}_2}}\cdot {m}_{\mathrm{Ni}{\left(\mathrm{O}\mathrm{H}\right)}_2}+\frac{M_{\mathrm{Ni}\mathrm{O}}}{M_{\mathrm{Ni}\mathrm{C}{\mathrm{O}}_3}}\cdot {m}_{\mathrm{Ni}\mathrm{C}{\mathrm{O}}_3}+\frac{M_{\mathrm{Ni}\mathrm{O}}}{M_{\mathrm{Ni}{\left(\mathrm{C}{\mathrm{H}}_3\mathrm{C}\mathrm{O}\mathrm{O}\right)}_2}}\cdot {m}_{\mathrm{Ni}{\left(\mathrm{C}{\mathrm{H}}_3\mathrm{C}\mathrm{O}\mathrm{O}\right)}_2}+{m}_{{\mathrm{C}}_{400}};\hfill \\ {}\hfill {C}_{400}=\frac{m_{\mathrm{Ni}}+\frac{M_{\mathrm{Ni}}}{M_{\mathrm{Ni}\mathrm{O}}}\cdot {m}_{\mathrm{Ni}\mathrm{O}}+\frac{M_{\mathrm{Ni}}}{M_{\mathrm{Ni}{\left(\mathrm{O}\mathrm{H}\right)}_2}}\cdot {m}_{\mathrm{Ni}{\left(\mathrm{O}\mathrm{H}\right)}_2}+\frac{M_{\mathrm{Ni}}}{M_{\mathrm{Ni}\mathrm{C}{\mathrm{O}}_3.}}\cdot {m}_{NiC{O}_3}+\frac{M_{\mathrm{Ni}}}{M_{\mathrm{Ni}{\left(\mathrm{C}{\mathrm{H}}_3\mathrm{C}\mathrm{O}\mathrm{O}\right)}_2}}\cdot {m}_{\mathrm{Ni}{\left(\mathrm{C}{\mathrm{H}}_3\mathrm{C}\mathrm{O}\mathrm{O}\right)}_2}.}{m_{400}}.\hfill \end{array}\right. $$

There, *m*_0_, *m*_120_, and *m*_400_ - masses of powder: initial, after drying in inert atmosphere at 120°C, and annealing at 400°C. *m*_H2O_, *m*_Ni_, *m*_NiO_, *m*_Ni(OH)2_, *m*_NiCO3_, *m*_Ni(CH3COO)2_, and *m*_C0_ - masses of water, nickel, and nickel oxide; hydroxy-, carbonate-, and acetate-containing precursors; and free carbon in initial powder. *m*_C400_ - mass of free carbon in powder after annealing in inert atmosphere at 400°C. *M*_NiO_, *M*_Ni(OH)2_, *M*_NiCO3_, *M*_Ni(CH3COO)2_ - molar masses of nickel oxide and hydroxy-, carbonate-, and acetate-containing precursors. *C*_120_, *C*_400_ - nickel total content in powders after drying in inert atmosphere at 120°C and annealing at 400°C.

For simplification of calculation of chemical composition, carbonate- and acetate-containing precursors have been accepted as pure NiCO_3_ and Ni(CH_3_COO)_2_, respectively, and calculated from the data of carbonate and organic carbon content.

For obtaining C_120_ and C_400_ nickel total concentration and masses m_120_ and m_400_ and m_C400_, powders were heated to 120°C and 400°C in argon atmosphere with a heating rate of 20°C/h. Drying/annealing time was 5 h.

Concentration of free carbon has been identified as a mass of insoluble residuals after dissolving the powders in HNO_3_ and boiling for 2 h. For determination of organic carbon solutions, after separation, the free carbon is mixed with 20 ml of 0.1 mol/l solution of K_2_Cr_2_O_7_ in concentrated H_2_SO_4_ and boiled for 2 h. Organic carbon content has been identified as the quantity of realized CO_2_. Carbonated carbon content has been determined as realized CO_2_ after dissolving concentrated H_2_SO_4_ powders and boiling for 1 h. Absorption of realized CO_2_ has been carried out by 0.1 mol/l aqua solution of BaCl_2_.

Chemical analysis of nickel total content and organic and carbonate carbon has been performed by photo-colorimetric method using double-beam spectrophotometer SQ4802 UV/VIS (Unico, Dayton, USA).

Thermogravimetric analysis has been carried out using a Derivatograph Q-1000 Paulik-Paulik-Erdey (MOM, Budapest, Hungary) in the regime of continuous heating from 20°C to 650°C with a heating rate of 10°C/min.

X-ray diffraction method (XRD) has been performed with using diffractometer DRON-3 in Cu Kα radiation. Diffraction peaks were identified using PDF-2 database (JCPDC-ICDD, 1999).

Phase analysis and particle size of powders have been investigated by X-ray diffraction method, transmission electron microscopy (TEM), and scanning electron microscopy (SEM). Powders’ pore structure has been determinated by low-temperature nitrogen-adsorption method.

Microscopic investigations of obtained powders have been carried out using high-resolution transmission electron microscope JEM-2100 F (Jeol, Akishima-shi, Japan) and scanning electron microscope EVO 50 XVP (Zeiss, Oberkochen, Germany). The particle size distributions have been obtained from measurement of 300 to 500 particles in SEM micrographs. Deviation in particle diameter measurements was 5 nm.

Low-temperature nitrogen-adsorption method has been performed using apparatus ASAP 2000 M (Accelerated Surface Area and Porosimetry System, Micromeritics Instrument Corporation, Norcross, USA). The range of equal pore size for this method was from 0.3 to 300 nm. Obtained isotherms have been calculated with the Barrett E.P., Joyner L.S., Halenda P.P (BJH) theory. Before carrying out the analysis, samples have been dried in vacuum at temperature 170°C.

## Results and discussion

### Annealing temperature effect on powder composition and particle size

**Figure 1:**
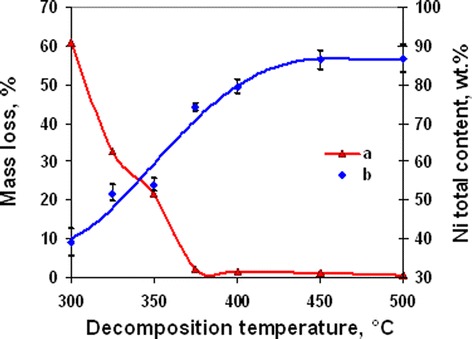
**Powder mass loss (curve a) and nickel total content (curve b) dependence on annealing temperature (annealing time 30 min).** Mass loss (curve a) and nickel total content (curve b) dependence on annealing temperature for nickel acetate hexaammine decomposition products (annealing time 30 min).

**Figure 2:**
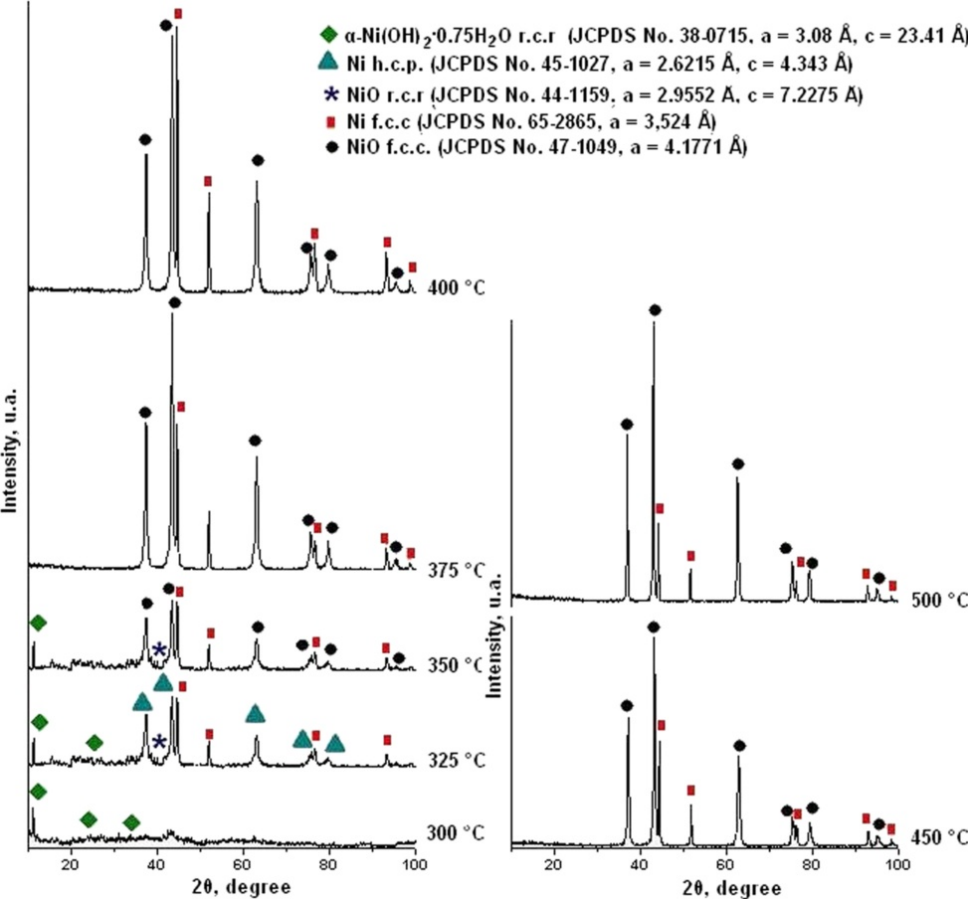
**X-ray diffraction patterns of nickel acetate hexaammine decomposition products obtained at different temperatures (annealing time 30 min).**

**Figure 3:**
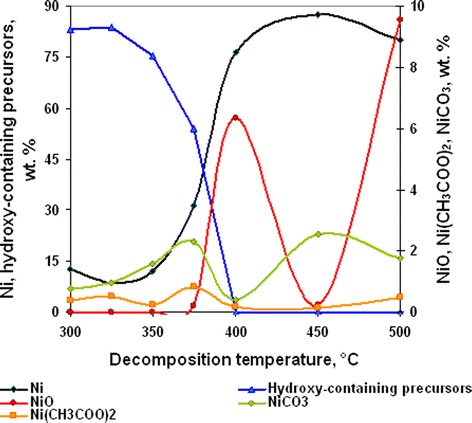
**Powder chemical composition dependence from annealing temperature for nickel acetate hexaammine decomposition products (annealing time 30 min).**

In accordance with thermal (Figure 1 curve a) and chemical (Figure 1 curve b) analyses, the degree of initial complex decomposition depended on the annealing temperature. Almost complete decomposition of the complex was observed at temperature range 375°C to 500°C and accompanied with the formation of Ni and NiO mixture. At lesser temperatures, duration of annealing was insufficient for full decomposition. In accordance with XRD data (Figure 2), powders contained some amount of hydroxy-containing precursors. The start of nickel- and nickel-oxide-phase formation was observed at 325°C. But chemical analysis of powder composition shows that on its own account NiO phase was formed at temperatures 375°C to 500°C only (Figure 3). In our opinion, this fact can be explained with presence of some amount of coordinated ammonia in the oxide phase because of incomplete complex decomposition at annealing temperature 325°C to 375°C.

Increase of annealing temperature from 300°C to 400°C led to the rising of Ni and NiO content in powder (Figure 3). But at higher temperatures, clear dependence of decomposition temperature on powder composition was absent. This fact can be explained with analysis of dependence of temperature on precursor composition (Figure 3). It is known from literature [40-42] that decomposition of nickel acetate hydrates in air occurs in three stages. At the first stage that is observed at 118°C to 137°C, some amount of acetate ions evaporates as acetic acid and formation of nickel hydroxy-acetates is observed. In this stage, increase of heating rate leads to a rise of degree of acetate ion removal [41]. In the next stage, at 345°C to 350°C, decomposition of nickel hydroxy-acetates to carbonate-containing compounds occurs. The last stage of decomposition is observed at 365°C. In this stage, carbonate-containing compounds decompose with formation of mixture of metal and oxide crystalline phases.

Figure 3 shows that decomposition of nickel acetate ammonia complex at temperatures from 300°C to 400°C occurred with destruction of hydroxy-containing precursors to metal nickel mainly. Also, formation of some amount of carbonate- and acetate-containing precursors is observed at this temperature range. Decomposition of carbonate-containing precursors to NiO begins at 400°C only. Increase of carbonate-containing precursor content at decomposition temperatures 450°C to 500°C was connected with the enhancement of acetate ion destruction to carbonate. This process was accompanied with decrease of decomposition rate and yield of NiO. Rise of NiO content in powder at 500°C was connected with the start of oxidation of metal nickel to NiO and does not corresponded with increase of decomposition rate of carbonate-containing precursors (Figure 3).

**Figure 4:**
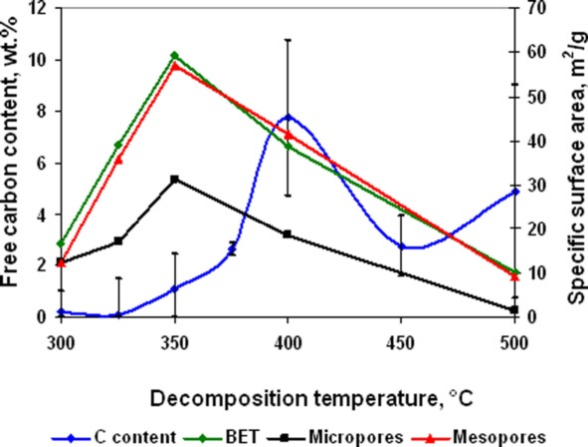
**Free carbon content and specific surface area dependence from annealing temperature for nickel acetate hexaammine decomposition products (annealing time 30 min).**

Change of decomposition mechanism led to increase of free carbon content in powders (Figure 4). Combination of this fact with decrease of specific surface area (Figure 4) of powders allowed the expectation that increase of free carbon content is connected with the inhibition of acetic acid evaporation because of the growth of powder particle size. The maximal volume of specific surface area at 350°C (Figure 4) indicates that this annealing temperature is more optimal for obtaining powders with minimal particle size and free carbon content.

**Figure 5:**
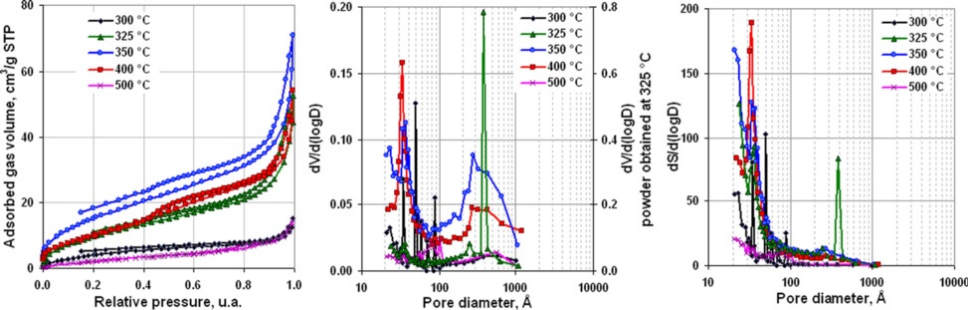
**Adsorption isotherms, differential volume, and area pore distributions of powders obtained at different annealing temperatures (annealing time 30 min).**

Analysis of the pore structure of obtained powders (Figure 5) shows that precursors’ decomposition to Ni and NiO at 350°C and 400°C, respectively, resulted in formation of mesoporous slit pore structure [43]. Low-pressure hysteresis in isotherms of powders obtained at 300°C and 325°C indicates that these products had an amorphous nonporous structure [43]. At temperature 300°C, the nonporous structure had molecular apertures on the surface. Increasing the annealing temperature to 325°C led to the formation of solid nonporous amorphous particles with mean size 37 to 38 nm. The process of decomposition of intermediate amorphous structure with formation of Ni and NiO crystalline particles (Figure 5 350°C, 400°C, respectively) was accompanied with the appearance of pores with mean size 3 to 4 nm and steps in adsorption isotherms which indicate existence of slit pore structure [37]. The linkage of isotherm adsorption-desorption legs that was observed for powders obtained at 400°C and 500°C shows the formation of a mixture of two phases in powder. Increasing mean pore size at annealing temperature 500°C, decomposition corresponded with growth of powder particles.

**Figure 6:**
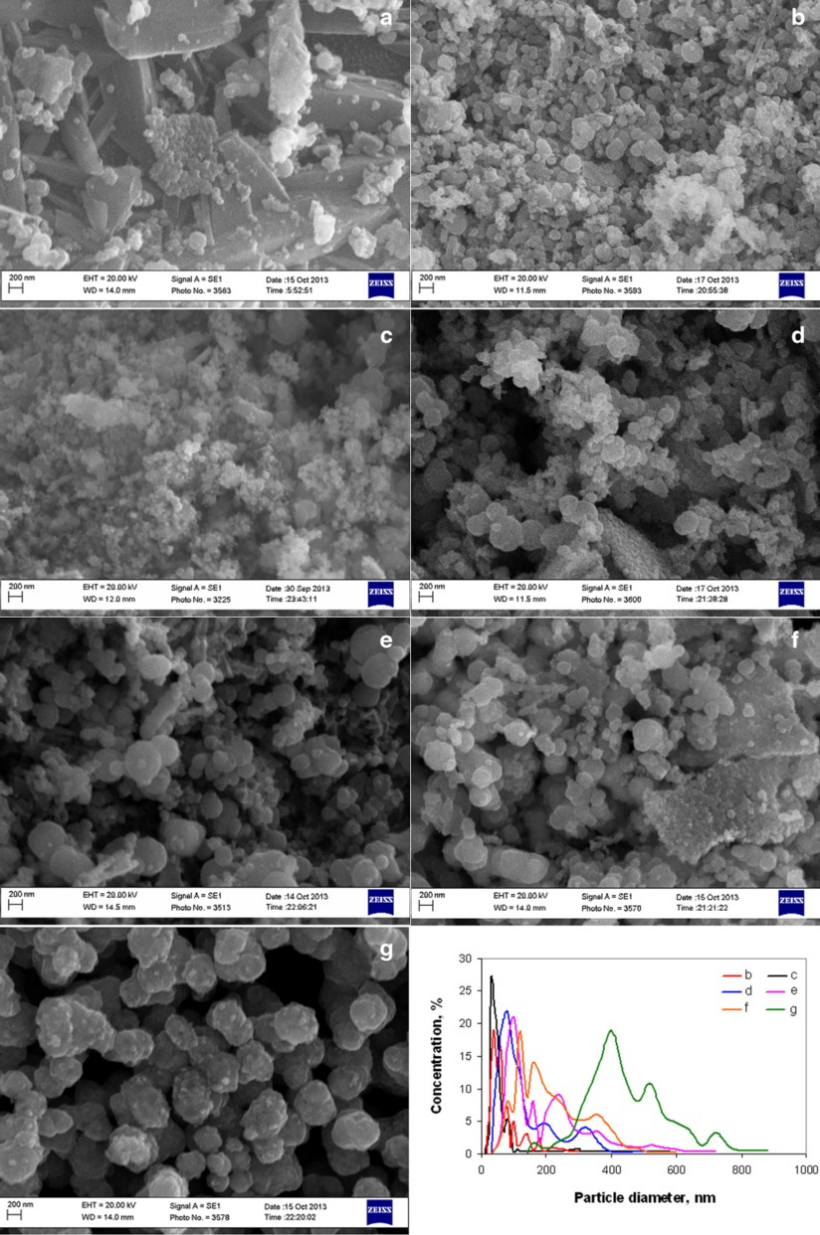
**SEM micrographs and particle size distribution of powders obtained at different annealing temperatures. (a)** 300°С, **(b)** 325°С, **(c)** 350°С, **(d)** 375°С, **(e)** 400°С, **(f)** 450°С, and **(g)** 500°С.

**Figure 7:**
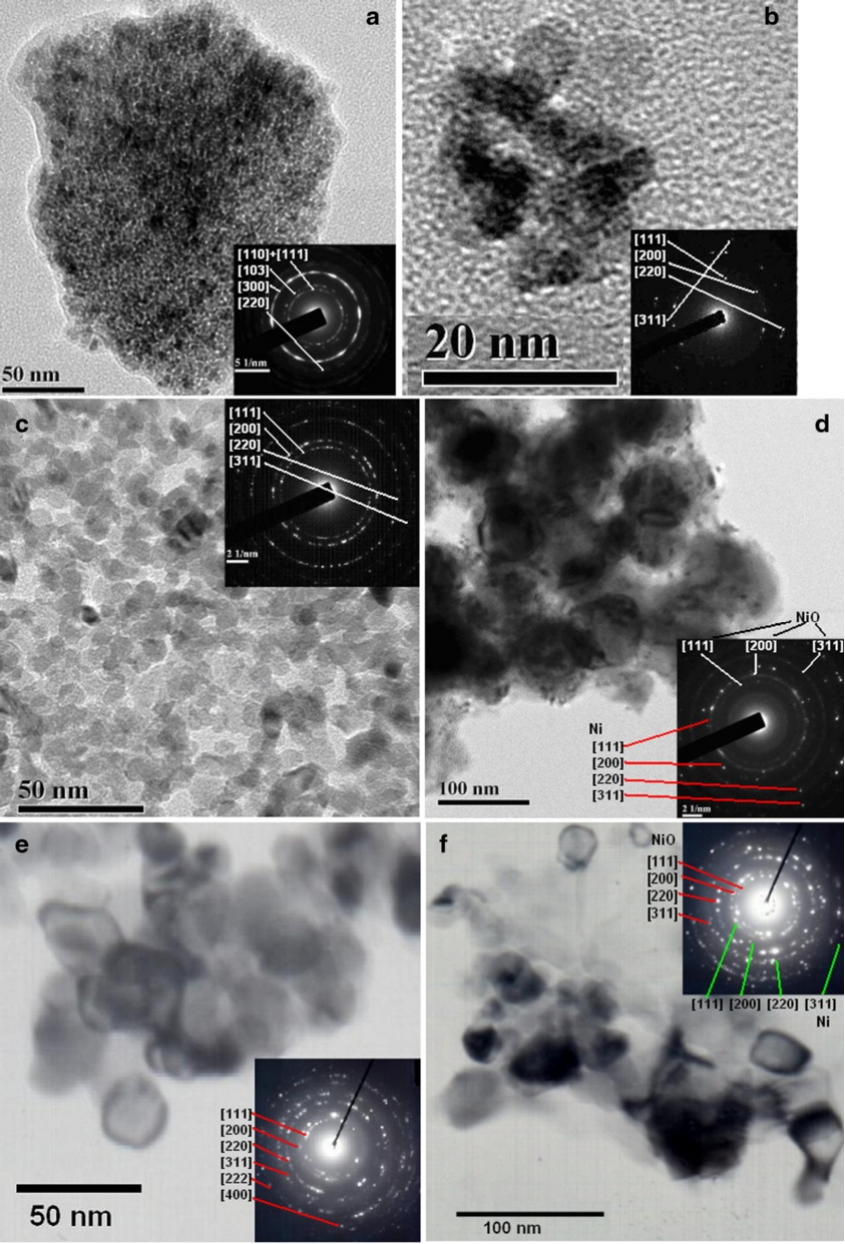
**TEM micrographs and particle size distribution of powders obtained at 350°С (a, b), 400°С (c, d), and 500°С (e, f). (a)** hydroxy-containing precursor (α-3Ni(OH)_2_ · 2H_2_O JCPDC No. 22–0444, h.c.p., *a* = 5.34 Å, *c* = 7.50 Å), **(b)** NiO (JCPDC No. 47–0444, f.c.c., *a* = 5.34 Å), **(c)** NiO (JCPDC No. 47–0444), **(d)** Ni/NiO(Ni JCPDC No. 65–2865, f.c.c., *a* = 3.524 Å, NiO - JCPDC No. 47–0444), **(e)** NiO (JCPDC No. 47–0444), **(f)** Ni/NiO (JCPDC No. 65–2865, JCPDC No. 47–0444).

Changing of pore structure of powders corresponded with changing of particle size obtained by SEM and TEM (Figures 6 and 7). Particle size distribution obtained by SEM micrographs has been presented for comparing of nanoparticles sizes from TEM. In accordance with our previous investigation [34,36], obtained powders resented by nanoparticles with two different sizes - NiO and Ni in the one hand. In the other hand, Ni nanoparticles mainly separated in certain particles and NiO nanoparticles connected in aggregates. Analysis of particle size distribution of suspensions obtained from powders [36] showed that aggregates of nanoparticles do not separate in certain particles. In this case, minimal grain size (minimal size of particles that was used for forming of multilayered ceramic capacitor (MLCC) electrode) corresponds to minimal aggregate size, and particle size distribution obtained from SEM is more promising for estimation of powder suitability for MLCC electrode manufacturing.

In accordance with SEM micrographs at temperature 300°C, the product was represented as solid flakes (Figure 6a), which were destroyed in separate agglomerates of nanoparticles at 325°C (Figure 6b.) with a minimal size of 30 to 50 nm (Figure 6 curve b). Full flake destruction in agglomerates with mean size 30 to 40 nm and mainly monomodal particle size distribution (Figure 6c, Figure 6 curve c) was observed at 350°C. Increase of annealing temperature to more than 350°C led to a rise of nanoparticle agglomerates size (Figure 6 curves d, e, and f). And at 500°C (Figure 6g, Figure 6 curve g), nanoparticles sintered in solid aggregates.

Particle size of 5 nm for nickel hydroxy-containing precursors was unchanged with temperature (Figure 7a). Mean nanoparticle size of Ni depended on annealing temperature and has grown from 40 to 60 to 40 to 70 nm at temperatures 400°С and 500°С, respectively (Figure 7d, f). Mean nanoparticle size of NiO increased with temperature rising from 5 nm at 350°С (Figure 7b) to 20 to 25 nm at 500°С (Figure 7e).

Thus, annealing temperature determinates the degree of initial complex decomposition, free carbon content in powder, mean nanoparticles, and agglomerate size. In accordance with the results of experiments, temperature 350°C has been considered the more promising one for obtaining of Ni/NiO nanopowders with minimal particle size and free carbon content.

### Annealing duration effect on powder composition and particle size

Since the powder obtained at temperature 350°C contained considerable concentration of precursor, the next stage of investigation was determination of the effect of decomposition time on powder composition and particle size.

**Figure 8:**
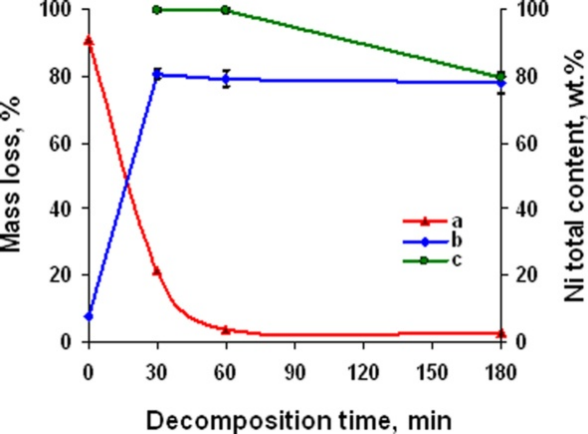
**Powders’ mass loss (curve a) and nickel total content (curves b, c) at various annealing duration at 350°C.** (curve b) Initial powder; (curve c) decomposition in inert atmosphere.

**Figure 9:**
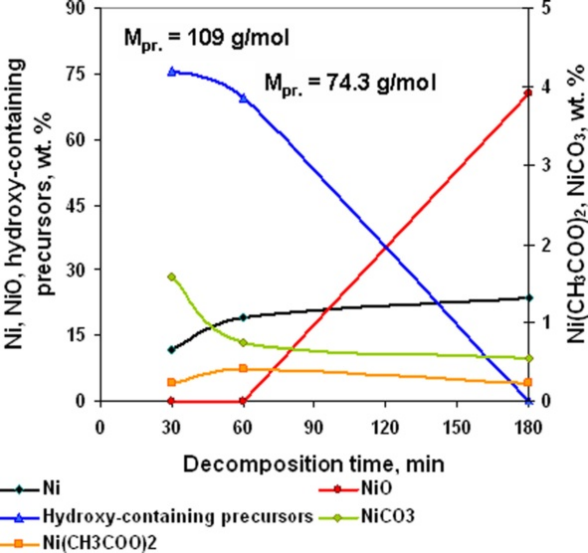
**Chemical composition dependence from annealing duration for nickel acetate hexaammine decomposition products obtained at 350°C.**

**Figure 10:**
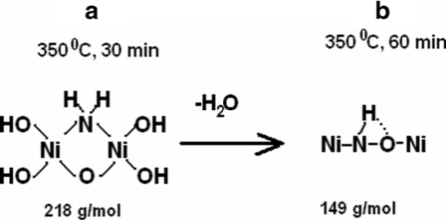
**Structure of hydroxy-containing precursors. (a)** structure (OH)_2_Ni(NH_2_)ONi(OH)_2_; **(b)** structure NiO(H)NNi.

Thermal (Figure 8 curve a) and chemical (Figure 8 curve b) analyses show that almost complete decomposition of nickel acetate ammine at annealing temperature 350°C was observed at 60 min of decomposition. But chemical analysis of powder composition (Figure 9) shows that complete decomposition of hydroxy-containing precursors was observed at annealing duration of 180 min and accompanied with primary formation of nickel oxide. In accordance with precursor component analysis (Figure 9), the concentration of carbonate- and acetate-containing precursors was primarily invariable at the investigated range of decomposition duration, and complex decomposition occurred with the destruction of hydroxy-containing compounds. Since decomposition of hydroxy-containing precursors which are formed at decomposition duration 30 and 60 min resulted in primary formation of NiO, it can be supposed that their composition is too close to nickel hydroxide. But this assumption is correct for precursor obtained at 30 min of decomposition only. In the one hand, molar mass of precursor if their content is only 1 atom of Ni was 109.2 and 74.3 g/mol for annealing time 30 and 60 min, respectively. In the other hand, decomposition of these precursors in inert atmosphere resulted in formation of Ni only (Figure 8 curve c), and in air, they decompose with the formation of mixture of Ni and NiO (Figure 9). Summarizing these facts makes it possible to expect that molecules of precursors contain ammonia or its derivatives. And in this case, approximate composition of precursors corresponds to structures (OH)_2_Ni(NH_2_)ONi(OH)_2_ (Figure 10 structure a) at 30 min of annealing and at 60 min - NiO(H)NNi (Figure 10 structure b). The molar mass of these precursors is 218 and 149 g/mol to 2 atoms of Ni in molecule. In this case, the precursor obtained at annealing duration 60 min corresponded to the final stage of decomposition and may indicate formation of Ni and NiO mixture with high volume of chemisorbed ammonia in pores. So the annealing duration of 60 min can be considered as the optimal one for obtaining Ni/NiO nanopowders.

**Figure 11:**
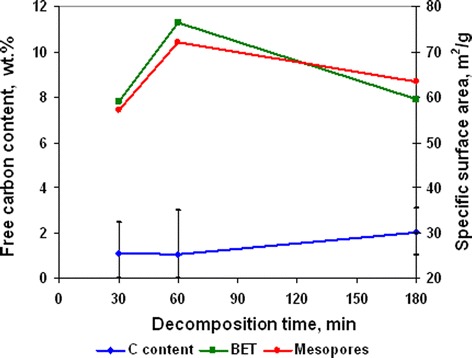
**Free carbon content and specific surface area dependence from annealing duration at annealing temperature 350°С.**

**Figure 12:**
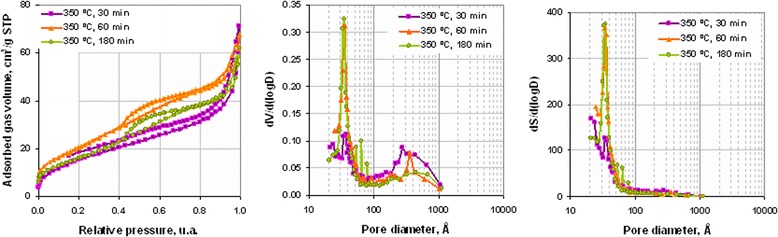
**Adsorption isotherms, differential volume, and area pore distributions of powders obtained at 350°С with different decomposition duration.**

**Figure 13:**
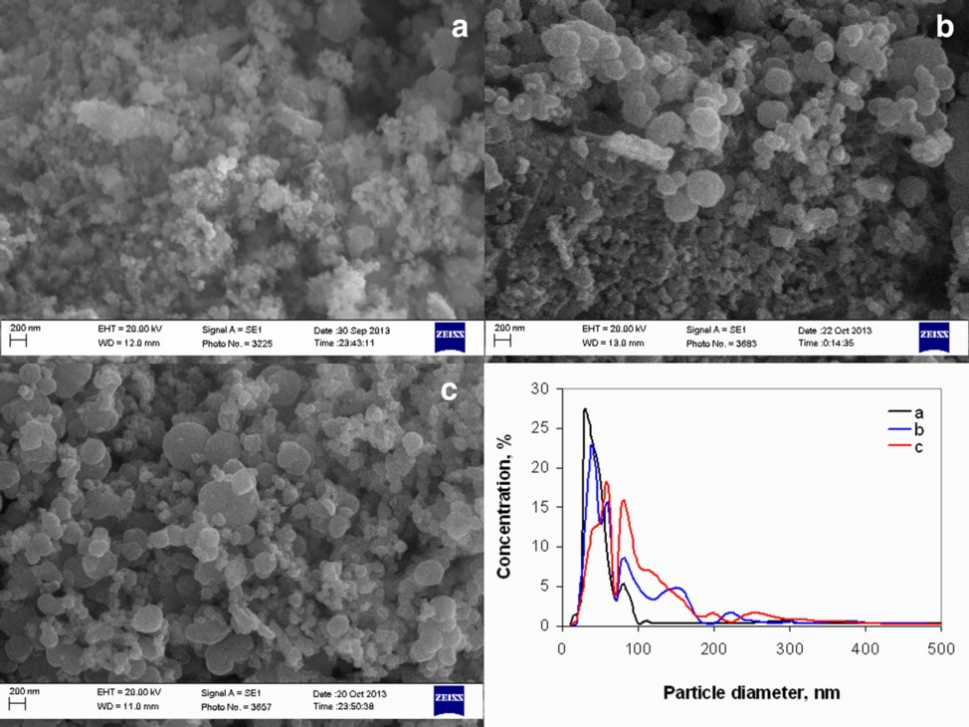
**SEM micrographs and particle size distribution of powders obtained 350°С.** annealing duration **(a)** 30, **(b)** 60, **(c)** – 180 min.

Free carbon content near 1 wt. % was invariable at the investigated range of decomposition duration (Figure 11). Specific surface area of powders increased at the first stage (Figure 11) of decomposition due to desorption of gases from pore surface. But increase of annealing duration from 60 to 180 min (Figure 11) led to some decrease of specific surface area. Change of powder pore structure (Figure 12) shows that increase of decomposition time from 30 to 60 min led to decreasing pore volume with mean size 20 to 50 nm and rising pore volume with size 3 to 4 nm. Thus, the initial increase of specific surface area (Figure 11) was generated by precursor decomposition with the forming of Ni and NiO. In accordance with SEM data (Figure 13b, Figure 13 curve b), this process was accompanied with the formation of nanoparticle agglomerates with a growth of 40, 60, and 80 nm and decreasing content of agglomerates with a size of 30 to 40 nm. Increase of annealing duration to 180 min led to a rise of agglomerates content with a mean size of 60 and 80 mn (Figure 13c, Figure 13 curve c). This process was accompanied with the appearance of discrete pore size distribution (Figure 12) that indicates the beginning of agglomerates growing because of their sintering during afterreduction of NiO nanoparticles to Ni by residuals of ammonia and organic compounds on agglomerate surface.

Thus, annealing temperature determinates degree of initial complex decomposition, free carbon content in powder, mean nanoparticles, and agglomerate size. In accordance with results of experiments, temperature 350°C has been considered the more promising one for obtaining of Ni/NiO nanopowders with minimal particles size and free carbon content.

### Ammonia content effect on powder composition and particle size

In the next stage of investigation, the effect of ammonia content on powder composition and particle size has been determinated.

**Figure 14:**
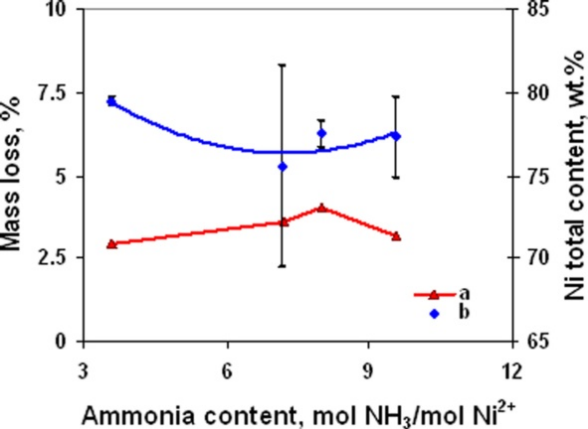
**Powders’ mass loss (curve a) and nickel total content (curve b) at various ammonia content in complex (350°C, 60 min).**

**Figure 15:**
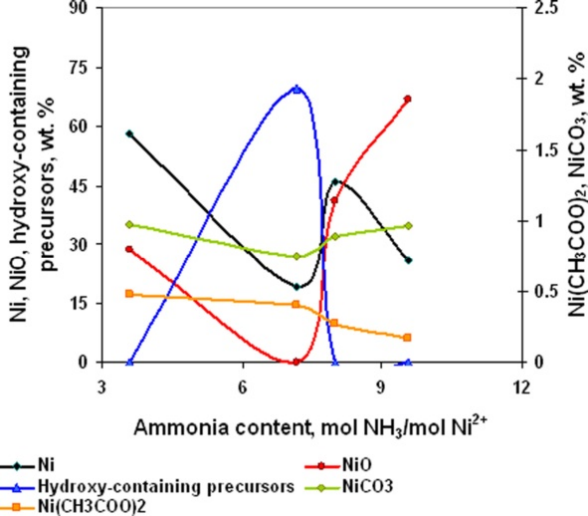
**Chemical composition dependence from ammonia content for powders obtained at 350°C (annealing time 60 min).**

Thermal (Figure 14 curve a) and chemical (Figure 14 curve b) analyses show that ammonia content in the initial complex did not effect on the rate of decomposition. But component composition of powder (Figure 15) permits the separation of the concentration range of 8 to 9.55 mol NH_3_/mol Ni^2+^ as optimal. Changing of initial complex composition did not effect on content of acetate- and carbonate-containing precursors. The low content of precursors in the powder with initial ammonia concentration of 3.6 mol/mol Ni^2+^ (Figure 15) permits the supposition that decomposition of the complex in this case is similar to decomposition of the hydrate of nickel acetate with as it has been noted previously primary full decomposition to mixture of Ni and NiO at temperature 350°C.

**Figure 16:**
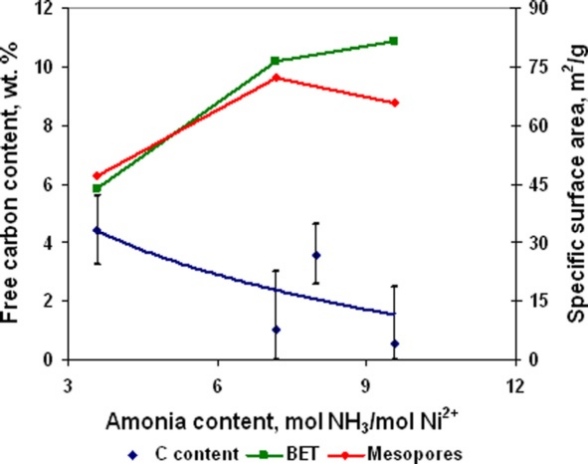
**Powders’ free carbon content and specific surface area at various ammonia content in complex (350°C, 60 min).**

**Figure 17:**
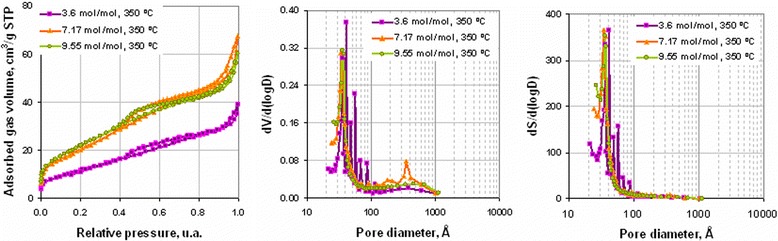
**Powders’ adsorption isotherms, differential volume, and area pore distributions at various ammonia content in complex (350°C, 60 min).**

**Figure 18:**
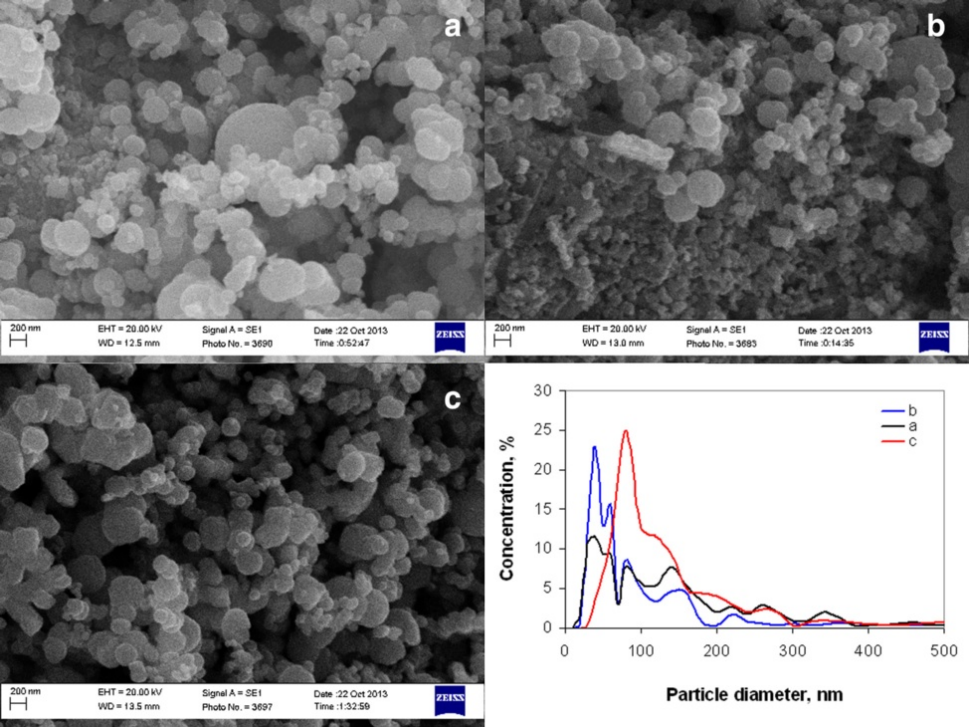
**SEM micrographs and particles size distribution of powders obtained 350°С and 60 min.** Ammonia content in initial complex **(a)** 3.6 mol /mol Ni^2+^; **(b)** 7.17 mol/mol Ni^2+^; **(c)** 9.55 mol /mol Ni^2+^.

Dependences of ammonia content on free carbon concentration and specific surface area (Figure 16) show that ammonia concentration of 9.55 mol/mol Ni^2+^ was optimal to obtain powders with minimal particle size and free carbon content. Analysis of powder pore structure (Figure 17) shows that increase of ammonia content in the initial complex permitted the decrease of mean pore size and correspondingly the decrease of mean particle size in powder. At the same time, at ammonia content in the initial complex of 3.6 mol/mol Ni^2+^, the discrete pore size distribution was observed which indicates that process of decomposition occurred irregularly. In accordance with SEM (Figure 18a, Figure 18 curve a), such irregular decomposition was accompanied with the formation of product with wide multimodal agglomerate size distribution. Increase of ammonia content in the initial complex (Figure 18) permitted to narrow the range of agglomerate size distribution with the formation of primary monomodal distribution at NH_3_ concentration 9.55 mol/mol Ni^2+^ (Figure 18c, Figure 18 curve c).

Changing in nanoparticle agglomerate size is generated by two effects of NH_3_ on powder. In the one hand, ammonia acts as a dispersing agent for nanoparticles and aggregates of nanoparticles. In the other hand, annealing temperature of 350°C is sufficient for start of reduction of NiO on the particle surface to Ni by NH_3_ but insufficient for sizable powder deaggregation as a result of intensive gaseous desorption. Thereby, in concentration range 3.6 to 7.17 mol NH_3_/mol Ni^2+^, ammonia primarily effects as a dispersing agent of aggregates. But further increase of concentration to 9.55 mol NH_3_/mol Ni^2+^ leads to intensification of the reduction processes on the particle surface because of simultaneous decreasing of nanoparticle size and increasing of ammonia content on pores.

Thus, ammonia concentration in the initial complex at annealing temperature 350°C and duration of 60 min has a direct influence on size and size distribution of nanoparticle agglomerates. In accordance with the results of experiments, the most optimal conditions considered for Ni/NiO nanopowders obtained by thermal decomposition of nickel acetate ammines were ammonia concentration in initial complex 9.55 mol /mol Ni^2+^, annealing temperature of 350°C, and duration of 60 min. The powder obtained at these conditions contained 0.59 wt. % of free carbon and had the most uniform particle size distribution.

## Conclusions

In the paper, effect of annealing temperature and duration and ammonia content in initial complex on composition and particle size of Ni/NiO nanopowders obtained by thermal decomposition of aqua solutions of nickel acetate ammines in air has been investigated.

It has been determinated that at temperature range of 300°C to 500°C, decomposition of nickel acetate ammines occurred with primary formation of hydroxy-containing precursors which approximate the composition Ni_x_(OH)_y_O_z_(NH_3-n_)_p_. Presence of ammonia and its derivatives in precursor molecule results in possibility of formation Ni and NiO phases during the decomposition process. Also, at this temperature range, formation of some amount of acetate- and carbonate-containing precursors was observed. Content of acetate- and carbonate-containing precursors depended on annealing temperature and was 0 to 1.64 wt. % and 0 to 3 wt. %, respectively.

Precursor decomposition with formation of crystalline Ni and NiO accompanied the formation of products with slit pore structure with mean pore size of 3 to 4 nm. Products of decomposition were represented as agglomerates of nanoparticles of Ni, NiO, and hydroxy-containing precursors. Mean agglomerate size depended on annealing temperature and duration and ammonia content in the initial complex. Increase of decomposition temperature from 300°C to 350°C led to decrease of agglomerate size because of destruction of initial nonporous amorphous structure of precursors. At temperature range of 350°C to 500°C and annealing duration of 60 to 180 min at 350°C, the rise of temperature and time of decomposition resulted in increase of nanoparticle agglomerate size because of sintering and after reduction processes. Increase of ammonia content from 3.6 to 7.17 mol/mol Ni^2+^ led to a decrease of agglomerate size in powder. NH_3_ concentration rising from 7.17 to 9.55 mol/mol Ni^2+^ resulted in increase of mean agglomerate size from 40 and 60 nm to 80 nm in the one hand. In the other hand, it permitted obtaining a product with primary monomodal agglomerates size distribution.

Mean nanoparticle size of hydroxy-containing precursors was invariable with ammonia concentration in the initial complex, annealing temperature, and duration and has grown 5 nm. Mean nanoparticle size of Ni depended on annealing temperature and has grown from 40 to 60 to 40 to 70 nm at temperatures of 400°С and 500°С, respectively. Mean nanoparticle size of NiO increased with the temperature rising from 5 nm at 350°С to 20 to 25 nm at 500°С.

The optimal condition for obtaining Ni/NiO nanopowders with low content of free carbon (0.59 wt. %) and primary monomodal agglomerate size distribution was considered to be the annealing temperature of 350°С, time of decomposition of 60 min, and ammonia content in initial complex 9.55 mol/mol Ni^2+^.

## Abbreviations

MLCC: multilayered ceramic capacitor
SEM: scanning electron microscopy
TEM: transmission electron microscopy
